# Management of the knees in arthrogryposis

**DOI:** 10.1007/s11832-015-0695-3

**Published:** 2015-10-26

**Authors:** Eva Pontén

**Affiliations:** Department of Pediatric Orthopaedic Surgery, Astrid Lindgren Children’s Hospital, Karolinska University Hospital, 171 76 Stockholm, Sweden

**Keywords:** Arthrogryposis, AMC, Amyoplasia, Distal arthrogryposis, Pterygium, Knee

## Abstract

Arthrogryposis is defined as limited range of motion in three or more joints in two or more body parts. This article will describe treatment options for the arthrogrypotic knee. In all types of arthrogryposis, and in both extension and flexion deformities, very early treatment is favorable. Just after birth, traction and mobilization followed by serial casting could often greatly improve the range of motion. In the hyperextended knee, surgical lengthening of the extensor apparatus may be needed. Flexion deformities could be improved with temporary physeal arrest of the anterior distal femur by fixing two-hole plates over the physis on both sides of patella. The plates will result in a constrained growth of the anterior physis, and thus a very slow extension of the knee, which will give the nerves and vessels time to adjust. Pterygium, webbing of the knee joint, is a special subgroup that in selected mild cases could be treated with extensive surgical release of the webbing and orthotics. Arthrogrypotic knees can be treated with early reduction and maintenance with orthotics.

Arthrogryposis is defined as motion limitation of three or more joints in different parts of the body, so joints other than the knees are involved in arthrogryposis [1]. However, some children are born with involvement of the knees only. As the treatment follows the same principles, the following text will include both arthrogryposis and isolated involvement of the knees.

The motion limitations are flexion contractures, extension contractures, or both. In the past, arthrogryposis was treated by surgery alone, and approximately 6 surgeries were needed per child [2]. Following the introduction of daily passive stretching and orthotics, the number of surgeries per child declined to 3 per child, and this was even before Ponseti club foot treatment was introduced [3]. Now, with the Ponseti regimen, the mean number of surgeries needed per child is probably even less. Follow-up studies have shown that serial casting and orthotics are useful for maintaining the positions, and that muscle strength is functionally more important than lack of contractures. Therefore, the focus should be on preserving musculature [4, 5]. Carlson et al. found that walking is easier if the legs and feet have a good alignment and one-sided hip subluxation/dislocation is reduced. However, independence was not dependent on functional disability but on mental capacity [6].

The treatment aim is thus to obtain independence and optimal function in daily life. We need legs that are optimally positioned for walking, standing, and sitting. Feet function optimally when plantigrade, and walking is more stable if the hips are in the joint. A knee flexion contracture of >15° hampers walking, 110° of flexion is needed for bicycling, and knee hyperextension makes sitting problematic and walking difficult or impossible. 90° of knee flexion is desired for easy sitting. These goals are most readily achieved with treatment very early in life when the connective tissue is compliant and the non-calcified cartilaginous joints are elastic. Very early manipulations and orthotics, within hours/days after birth, may almost normalize joint and ligament configurations. Often serial casting is more effective than orthotics, as it is easier to obtain a tight and correct fit. The muscles should be activated early for optimal development of strength and mobility.

A knee contracture can have various causes, e.g., amyoplasia, distal arthrogryposis, pterygium, and other malformations. Contractures in amyoplasia are very resistant to treatment. Contractures in distal arthrogryposis often improve during the first months of life. After birth, manipulations and orthotics or serial casting can be performed. If the child also has club feet, Ponseti manipulations require the knee to be flexed so that abduction of the foot can be controlled.

## Flexion contracture of the knee

**Fig. 1:**
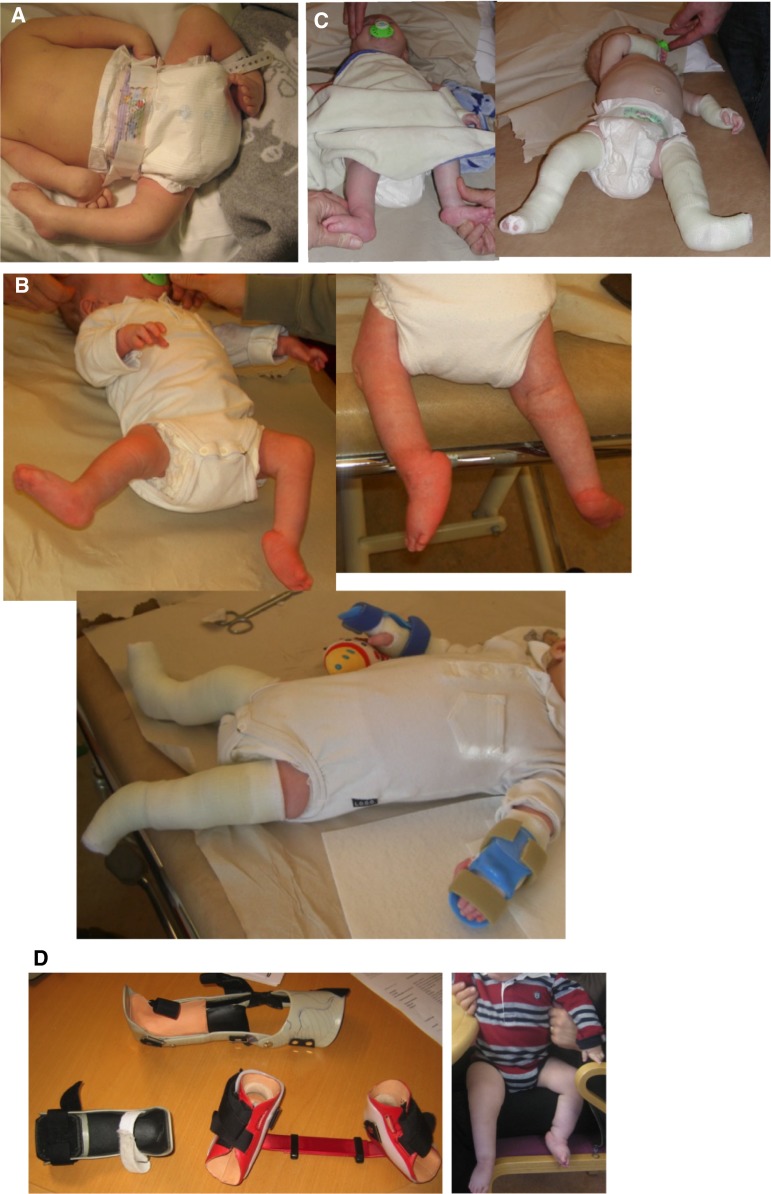

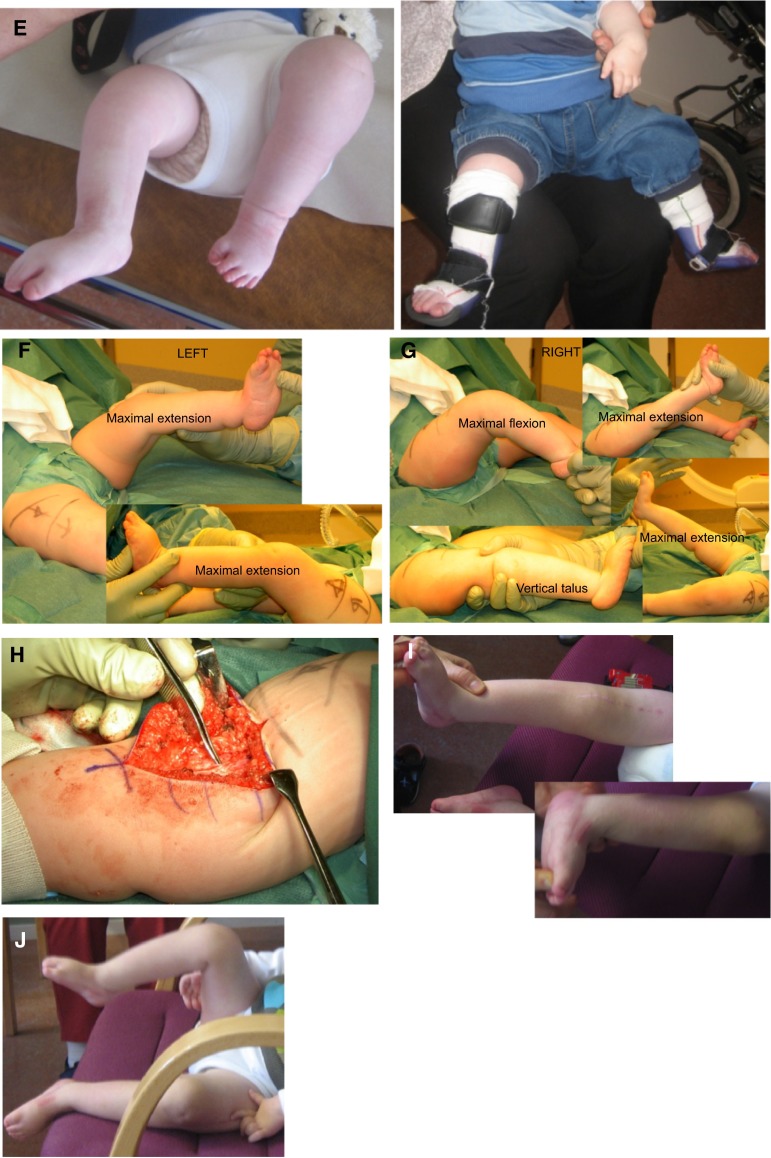
**a** Child a few days old with arthrogryposis (amyoplasia type). Right leg: congenital knee dislocation, abduction contracture of hip, vertical talus, and equinus of foot. Left leg: flexion contracture of knee, abduction contracture of hip, club foot. **b** Serial casting of knees and feet for almost 3 months. *Right* Gradual flexion of knee, inverted Ponseti for vertical talus. *Left* Gradual extension of knee, Ponseti manipulations, and casting for club foot. **c** We eventually obtained flexion of the right knee, a straight left knee, and less abduction of the hips. **d** Night-time: foot abduction brace. Day-time: *left* AFO, *right* hinged KAFO with elastic flexion. This eventually resulted in bending of the proximal tibia instead of flexion of the knee surgery. **e** Final result of serial casting. Right knee flexes 80°. Orthotics on day and night. **f** Surgery of all deformities in one session was planned; pre-op deformities of left leg are shown. **g** Pre-op deformities of right leg. **h** Right knee: distal part of the quadriceps was fibrotic. V–Y plasty of quadriceps was not sufficient for the distances needed. Fascia lata was used as an interposition graft. 90° of flexion. **I** *Right* Right leg: the quadriceps muscle extensively lengthened. Collateral ligaments partially divided. Previously vertical talus is now a normal arch in a plantigrade foot.* Left* Left leg: straight knee after extension osteotomy of distal femur. Previously club foot, now normal inclination of talus to navicular. No adductus, equinus, or varus. **J** Post-OP results. Child walks independently with open hinged KAFOs

Common to all congenital flexion contractures, when it is not possible to extend the knee to 0°, very early treatment gives better results. Just after birth, the connective tissue is very compliant, and a large proportion of the bone is not mineralized. Putting continuous pressure or tension on these tissues early can change the 3D structure of the joint. Doing it in a planned way may successfully improve the range of motion (Fig. 1a–j). Later, 8-plates can be applied over the ventral physis of the distal femur [7]. Palocaren et al. used 8-plates for 10 children (4–10 years old) with arthrogryposis and knee flexion deformities. They found that the knees got straighter and that the walking improved, especially when the contractures were <45° [8]. When applying 8-plates for flexion deformities of the knees, one has to make two arthrotomies, one on either side of the patella. A true lateral X-ray of the knee is mandatory when checking the position of the 8-plates during surgery, to ensure that the screws are inserted properly proximally and distally to the anterior distal femoral physis on each side of the patella. If it is difficult to obtain enough space anteriorly for the lateral 8-plate, it can instead be placed very anteriorly on the lateral facet of the distal femur. Care must be taken that no capsule is trapped by the 8-plate and screws. Postoperatively, only a bandage is put on the incisions, and full weight-bearing is allowed. Correction of the flexion deformity is checked every 6 months. When full extension is obtained, the screw furthest from the joint can be removed, “unlocking” the 8-plate. If the deformity recurs, a new metaphyseal screw can be applied to the plate that has been left in the knee, guiding the growth into extension a second time. After the physis is closed, the plates are removed.

Another option is to make a ventral closing wedge osteotomy of the distal femur, fixing it with a plate or with wires in the young child [9]. However, in the growing child, there is a recurrence rate of 1° per month. Not uncommonly there is a risk of neurovascular trauma with, e.g., hyperesthesia of the feet as a sign of nerve injury. This is not seen with the gradual effect of the 8-plates. Yet another option is surgical dorsal soft-tissue release with *z*-lengthening of the hamstrings and perhaps a dorsal capsulotomy. The drawback here, compared to 8-plate guided growth, is that lengthening of the hamstrings could result in weakness. Repeated serial casting is a nonsurgical option which has a limited risk for neurovascular complications.

## Pterygium syndromes

A pterygium of the knee is a web, i.e., a triangular membrane with shortness of skin and other soft tissues on the back of the leg. The inheritance is variable for the different multiple pterygium syndromes, and there are associated malformations and joint contractures [1]. In a popliteal pterygium, there is a taut fibrous cord originating from the ischial tuberosity and inserting into the dorsal part of the calcaneus. The sciatic nerve and the popliteal artery and veins are sometimes found in the web. The gastrocnemius is short and its origin on the dorsal distal femur is abnormally proximally situated. Laterally and medially, under the skin on the dorsal side of the knee joint there is fibrous webbing, often with the peroneal nerve weaved into the connective tissue web.

Shortly after a child with popliteal pterygium is born, stretching of the web by serial casting of the whole leg can be started, with careful molding of the cast around the feet. Early percutaneous tenotomy of the Achilles tendon will facilitate a better foot position, with less equinus of the calcaneus. After a few weeks, when no more improvement is being achieved, KAFO orthotics are made in the most extended position, and should be worn most of the day and night. If the knee contracture is >20°, surgery is planned at about 1 year of age.

**Fig. 2:**
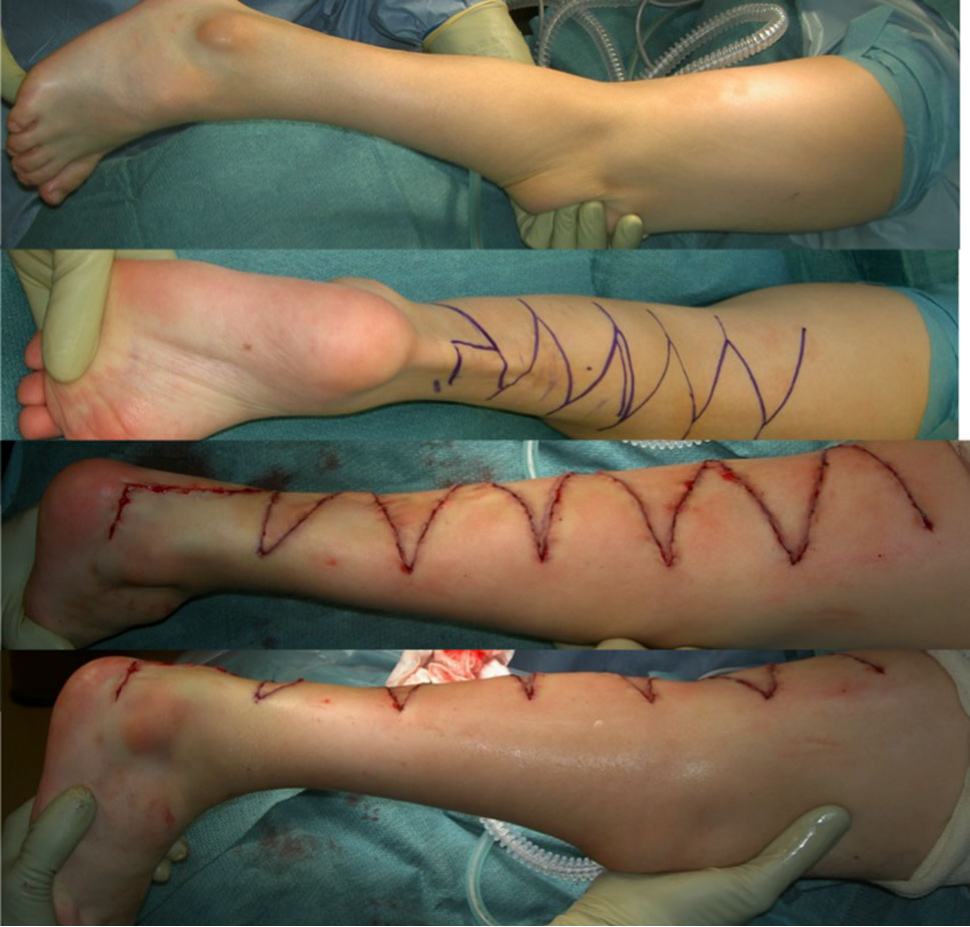
Pterygium: removal of the ischio-calcaneal string. The heel cord was lengthened, and dorsal capsulotomy and Y-V-plasty of skin was performed

For a mild pterygium, connected Y-V plasties of the skin over the length of the web are performed (Fig. 2). The ischio-calcaneal string is excised and the heel cord is lengthened. Meticulous dissection is performed so that all nerve and vessel branches are seen and freed from the webbed connective tissue, which is removed or cut so that knee extension is not hampered. The origin of the gastrocnemius on the femur sometimes needs to be released, and hamstring tendons may have to be *z*-lengthened. Dorsal capsulotomy of the knee joint may be performed so that the knee can be extended; aiming for full extension. The skin is then sutured with advancement of the triangular skin flaps so that the serial Ys will become Vs. This increases the length of the skin on the back of the leg. As there is usually stretching of the nerves and vessels when the leg is put in its new, extended position, the leg is casted in only slightly more extension compared to pre-surgery. The cast is then changed weekly, for about a month, casting in a more and more extended position each time until the leg is straight. If there is any kind of discomfort in the foot, the leg is casted again in a more flexed position until the discomfort (numbness, pain) has diminished. After the last cast has been worn for 1–2 weeks, a mold for a KAFO in an extended position is made. Until the KAFO is made, the leg is maintained in a straight leg cast. During the whole childhood and adolescence, nighttime KAFO is worn with the knee in extension and the ankle at 90°. Even then, recurrence of the flexion contracture may require more surgery. Then the Y-to-V plasties can be further advanced, increasing the length of the dorsal skin, and connective tissue hindering knee extension can again be removed or cut. The Achilles tendon needs to be re-lengthened.

It is possible to slowly stretch out the webbing of the knee using circular external fixators (e.g., Ilizarov frame) [10]. However, in a study by Kim et al., even though a tenotomy of the ischio-calcaneal band at the ischial tuberosity and a *z*-lengthening of the flexor hallucis longus was performed, a recurrence was observed at 2 years [11]. Other studies have shown good short-term results but with recurrence [12].

Arthrodesis of the knee in a straight position has been reported as a way of making it easier to ambulate short distances standing, but it severely impairs sitting and the usage of a wheelchair [13].

## Congenital knee dislocation

A congenital extension contracture of the knee is referred to as a congenital knee dislocation, even though it is a true dislocation only in its most severe form. It may be caused by fibrosis of the rectus part of the quadriceps or impaired muscle activity with muscle imbalance due to muscle protein mutations or deficits of the neuromuscular pathway. The cause of the fibrosis is not known, but one hypothesis is that it is the result of a circulatory dysfunction resulting in a compartment syndrome in the muscle.

As the fetus has not been able to bend the knees, the collateral ligaments are short, and the cruciate ligaments will not have developed correctly. In severe cases there are no cruciate ligaments, the collateral ligaments are very short, and the tibial plateau is luxated anteriorly [14, 15].

**Fig. 3:**
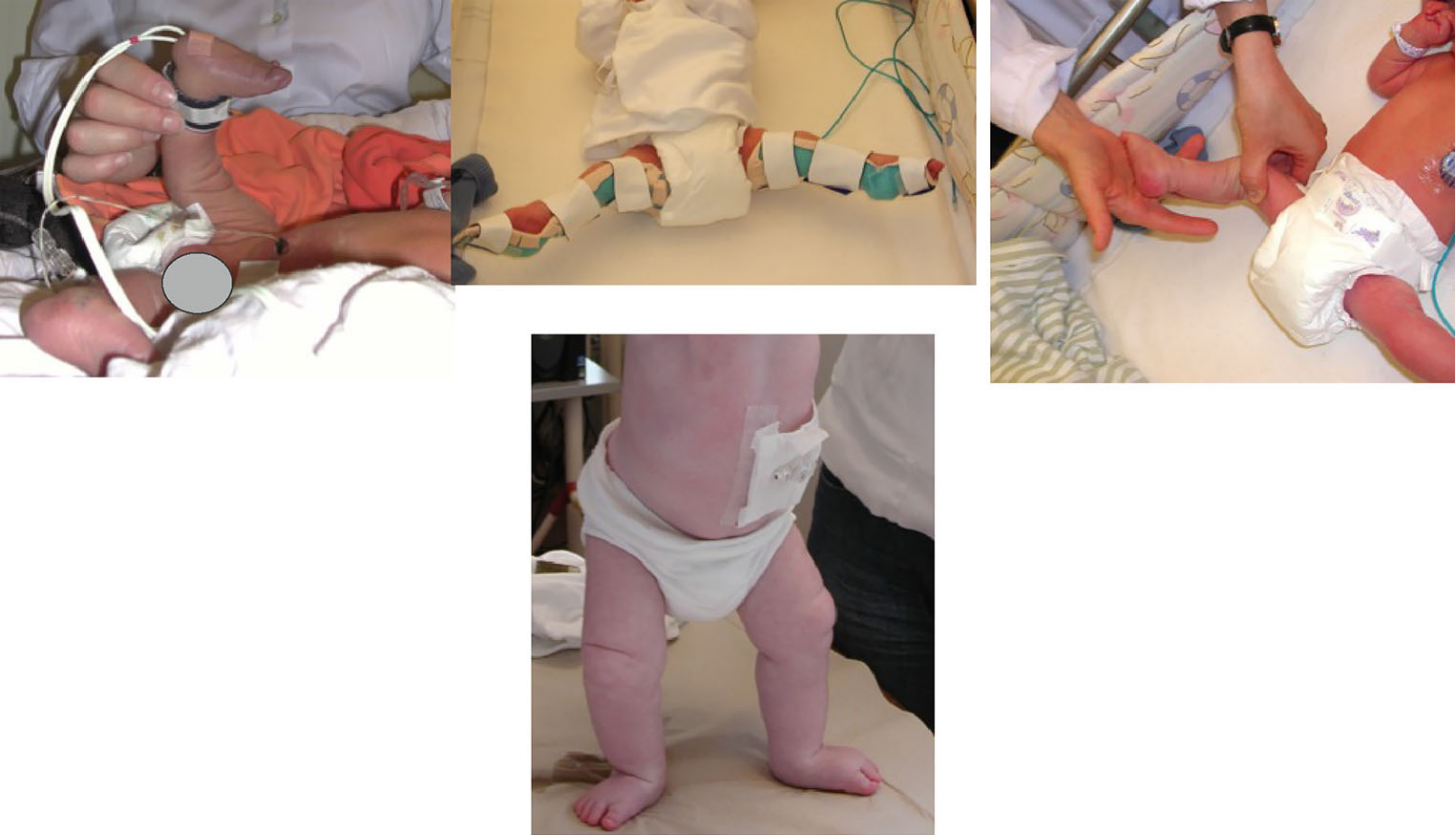
Bilateral congenital knee dislocation treated with manipulations and splints that were adjusted according to the increased ROM. No surgery was needed

Even though there are different causes of the extension contracture and the inability to bend the knee, all benefit from very early treatment, i.e., passive manipulations and serial casting (Fig. 3). For all children, the connective tissue is most compliant just after birth. The joints are cartilaginous early in life, and can more easily remodel during serial casting. This treatment regime will result in a better range of motion, in contrast to tendon lengthenings, which just move the center of the range of motion to another angle. When performing the manipulations, it is very important that a longitudinal stretch is first put upon the knee so that the femoral condyles are distracted from the tibial plateau. At that point, the knee can be flexed, avoiding a nutcracker phenomenon that would otherwise occur when the femoral condyles interfere with the tibia. Concomitant flexion of the hip will relieve tension on the rectus femoris, making it easier to flex the knee.

**Fig. 4:**
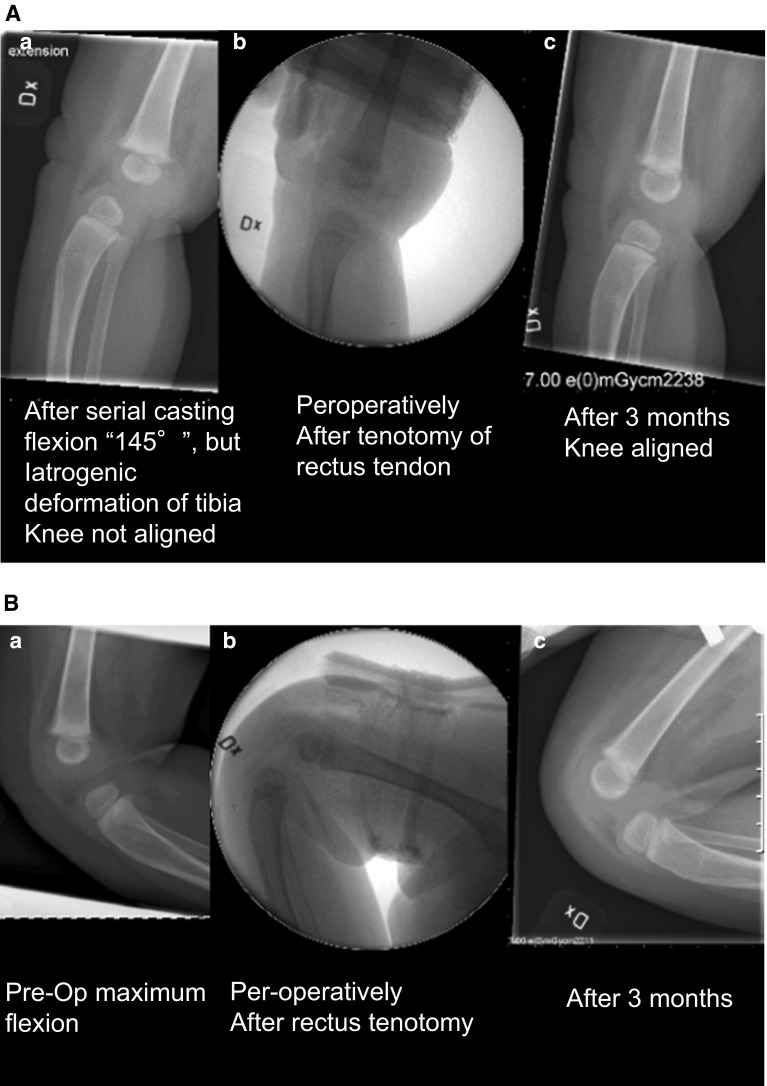
**A** *Left* After serial casting, flexion was 145° but there was an iatrogenic deformation of tibia. The knee was not aligned.* Middle* Peroperatively, after tenotomy of rectus tendon.* Right* After 3 months: knee aligned. **B** *Left* Same child, pre-op, maximum flexion.* Middle*: Peroperatively, after rectus tenotomy.* Right* Maximum flexion after 3 months
- *Early treatment with quadriceps tenotomy* If there is insufficient progress with serial manipulations and castings, a tenotomy of the rectus tendon can be performed, after which a cast is applied with the knee in a more flexed position. The tenotomy can be performed percutaneously [18] or with a mini-open technique [17]. If insufficient flexion is obtained during surgery, a release is made medial and lateral to the patella. If flexion remains limited, the whole anterior capsule is incised.
- If the above treatments do not suffice, a *V–Y plasty of the rectus* *+* *anterior capsulotomy* has been shown to allow flexion of the knee (Fig. 5).

Treatment of congenital knee dislocation can be categorized as follows:

- *Extremely early treatment*, within 24 h, as described by Chen et al. [16]. If treatment is started within 8 h, only 5 min of manipulation are required, but if manipulations start at 20–24 h after birth, about 20 min are needed. The knee is manipulated by repeatedly gently applying an anteriorly directed force to the distal femur and a posteriorly directed force to the proximal tibia until >90° of flexion is obtained. A splint is then applied so that the leg is kept in the maximally flexed position for 6–8 weeks, with the splint being changed every other week. X-ray confirms the reduction. If there is a concomitant hip luxation, a Pavlik harness could be used at the same time.
- *Early treatment*, within days, perhaps weeks. While the knee is stretched longitudinally, the distal femur is directed anteriorly and the proximal tibia is directed posteriorly. An orthosis or a splint is applied in the most flexed position (Fig. 3). Manipulations are performed every 5–7th day, with more and more flexion. X-ray may reveal a plastic deformation of the proximal tibia if too much pressure has been put on the tibia while the knee is still stiff and the tibial plateau cannot glide around the femoral condyle. This deformation may resolve with time but is an indication for surgery (Fig. 4a, b) [17].

**Fig. 5:**
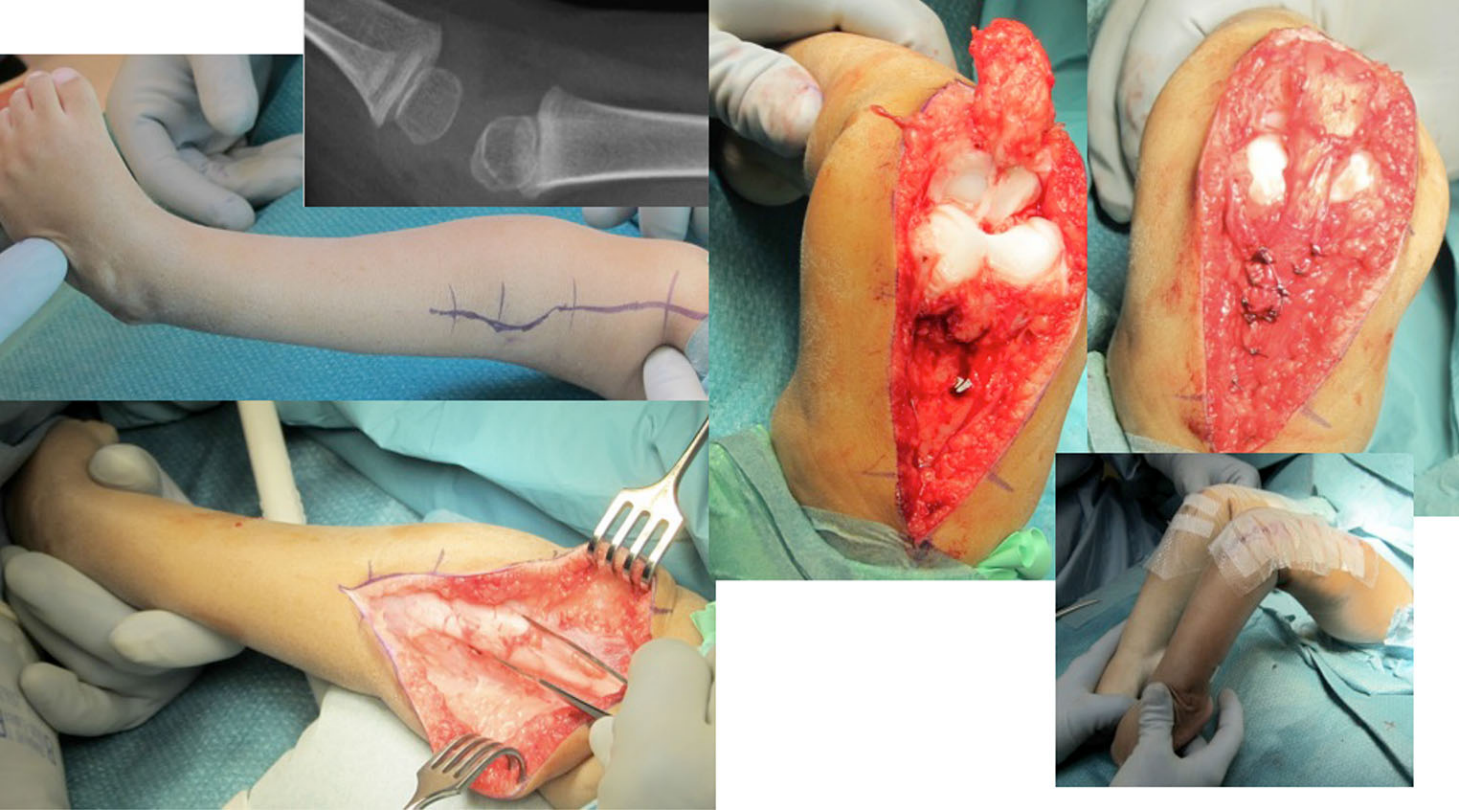
Child with extreme hydrocephalus, mental retardation, and bilateral congenital knee dislocations, pes equinovarus adductus, and the right hip dislocated. Extensive plasty of the extensor apparatus of the knees. The anterior part of the collateral ligaments was cut. Note that the cruciate ligaments were very rudimentary. The extensor apparatus is sutured in a V‐Y fashion so that the knee can be bent 90°
- Another option is *femoral shortening*, which will make both the extensors and flexors of the knee longer [15]. This will also make it easier to reduce a congenitally dislocated hip at the same time.
